# The Role of Mitochondrial Functional Proteins in ROS Production in Ischemic Heart Diseases

**DOI:** 10.1155/2016/5470457

**Published:** 2016-03-28

**Authors:** Haifeng Pei, Yi Yang, Heng Zhao, Xiuchuan Li, Dachun Yang, De Li, Yongjian Yang

**Affiliations:** ^1^Department of Cardiology, Chengdu Military General Hospital, Chengdu 610083, China; ^2^Department of Cardiology, Third Military Medical University, Chongqing 400042, China; ^3^Department of Ultrasonography, Chengdu Military General Hospital, Chengdu 610083, China

## Abstract

Ischemic heart diseases (IHD) have become the leading cause of death around the world, killing more than 7 million people annually. In IHD, the blockage of coronary vessels will cause irreversible cell injury and even death. As the “powerhouse” and “apoptosis center” in cardiomyocytes, mitochondria play critical roles in IHD. Ischemia insult can reduce myocardial ATP content, resulting in energy stress and overproduction of reactive oxygen species (ROS). Thus, mitochondrial abnormality has been identified as a hallmark of multiple cardiovascular disorders. To date, many studies have suggested that these mitochondrial proteins, such as electron transport chain (ETC) complexes, uncoupling proteins (UCPs), mitochondrial dynamic proteins, translocases of outer membrane (Tom) complex, and mitochondrial permeability transition pore (MPTP), can directly or indirectly influence mitochondria-originated ROS production, consequently determining the degree of mitochondrial dysfunction and myocardial impairment. Here, the focus of this review is to summarize the present understanding of the relationship between some mitochondrial functional proteins and ROS production in IHD.

## 1. Introduction

As the leading cause of fatality worldwide, ischemic heart diseases (IHD) give rise to widespread loss of cardiomyocytes and subsequent adverse cardiac remodeling [1]. More than 17 million people had succumbed to IHD in 2008, and the number was estimated to be 23.6 million by 2030 [2]. Ischemia starves cardiomyocytes of vital oxygen, resulting in severe or irreversible injury to heart [3]. Abundant lines of evidence indicate that reactive oxygen species (ROS) overload that originates from mitochondria is closely linked with the pathogenesis of cardiovascular diseases, such as atherosclerosis, myocardial infarction, and heart failure [4]. In myocytes, mitochondria comprise more than 30% cell volume and generate about 90% of the ATP [5]. Accordingly, mitochondria are also the major source of ROS in the cardiovascular diseases [5, 6]. Numerous experimental and clinical studies have shown that ROS accumulation is significantly exacerbated in the failing myocardium [7]. Chronic exposure of myocytes to ROS will lead to apoptosis, necrosis, and fibrosis, finally causing arrhythmias, impairment of excitation-contraction coupling, and cardiac remodeling [8–10]. Given that ROS affect virtually all aspects of cardiac myocyte physiology and that those lines of evidence support the notion that ROS are increased in IHD, the underlying mechanisms about ROS overproduction induced by mitochondrial abnormality should be further explored.

## 2. The Electron Transport Chain (ETC) Complexes and Uncoupling Proteins (UCPs)

Mitochondrial oxidative phosphorylation is the basic mechanism of ATP synthesis, in which the five enzyme complexes I–V in mitochondria are composed of multiple subunits. The respiratory chain, apart from energy production, plays a pivotal role in regulation of cardiac oxidative stress [11]. ETC damage will lead to a robust increase of ROS production in mitochondria [12]. In ischemic heart, oxygen delivery to myocardium is not sufficient to meet the demand of mitochondrial oxidation. Reintroduction of oxygen induced by reperfusion markedly enhances electron leakage from the ETC, resulting in overproduction of superoxide (O_2_^−^) and O_2_^−^-derived oxidants [12, 13]. Indeed, reduced activity of ETC subunits in heart failure patients has been confirmed independently of the etiology, notably of complex I [14], complex III [15], and complex IV [16]. Within the ETC, complexes I/III are regarded as the key contributors to ROS generation under stressful conditions [17, 18]. In the setting of myocardial ischemia/reperfusion (MI/R), oxidative injury of complex I can weaken complex interaction and enhance ROS production by complex III [19, 20]. Although the contribution of intact complex II seems to be negligible, some mutations in subunits of complex II have been linked to specific forms of cancer or neurodegeneration with increased ROS generation [21, 22]. Aldakkak et al. [23] revealed that pharmacologic blockade of electron transport attenuates ROS-induced cardiac injury. Consistently, nitrite can modulate mitochondrial resilience to reperfusion injury, perhaps via inhibiting complex I by posttranslational s-nitrosation to reduce ROS generation [24, 25]. Moreover, ROS scavenging ability may be impaired as an evident decrease of MnSOD activity in failing heart [26]. Coenzyme Q (CoQ) encompasses a collection of homologous molecules which consist of a benzoquinone ring linked to an isoprenoid side chain [27]. In the quinone form, CoQ serves as an electron carrier to transfer electrons in the mitochondrial ETC. In the quinol form (the reduced form of quinone), CoQ represents an efficient antioxidant in the body [28]. CoQ10 at high doses has also been proved to regulate gene transcription, including the genes important in lipid metabolism and the specific genes induced by ROS-sensitive intracellular pathways [29]. It is claimed that CoQ10 supplementation to obese rats attenuates age-related oxidative stress and preserves mitochondrial function in myocardium [30]. Moreover, CoQ10 deficiency is detrimental to the prognosis of chronic heart failure, and plasma concentration of CoQ10 may serve as an independent predictor of mortality [31]. Long-term supplementation of CoQ10 for patients with heart failure improves symptoms and reduces major adverse cardiovascular events [32].

In contrast, uncoupling proteins (UCPs) that are located in mitochondrial inner membrane exhibit opposite functions as complexes mentioned above. UCPs can reduce mitochondrial ROS production by dissipating the electrochemical gradient [33–36]. UCPs overexpression was found to salvage cardiomyocytes by preserving mitochondrial integrity [33, 36]. Specifically, mild to moderate mitochondrial uncoupling mediated by UCPs protects against MI/R injury by reducing ROS generation [37]. UCP1, characterized in brown adipocytes [38], has close relationship with endogenous CoQ to influence H^+^ transport [39]. In the setting of hypoxia/reoxygenation, UCP1 overexpression confers cardioprotection [33]. The presence of UCP1 mitigates reperfusion-induced damage, probably by reducing mitochondrial hyperpolarization [40]. Moreover, UCP2 overexpression can exert cardioprotection perhaps by preventing mitochondrial Ca^2+^ overload and attenuating ROS generation [41]. UCP3 has also been reported to play a critical role in cardioprotection during oxidative stress by suppressing detrimental ROS generation and maintaining myocardial high-energy phosphates [37]. In view of what was mentioned above, UCPs may be developed as protective targets for IHD patients.

## 3. Mitochondrial Dynamic Proteins

In order to adapt to stressful conditions, mitochondria dynamically undergo fission and fusion [42], which is basically regulated by some functional proteins [42, 43]. Mitofusin 1/2 (Mfn1/2) and optic atrophy 1 (Opa1) are known to induce mitochondrial fusion [44, 45], while dynamin-related protein 1 (Drp1) is identified to interact with fission protein 1 (Fis1) to promote mitochondrial fission [46, 47]. In mammalian cells, mitochondrial function is largely governed by mitochondrial fission and fusion which is an important factor for the integrity, structure, and function of healthy mitochondria. Abnormal fission or fusion will impair mitochondrial function, for example, resulting in overload of mitochondrial Ca^2+^, overproduction of free radicals, alteration of mitochondrial enzymatic activities, and reduction of ATP production [48].

Recent studies have highlighted the notion that defects in mitochondrial dynamics are relevant to various human disorders, including MI/R, heart failure, diabetes, and aging [49, 50]. Inhibition of mitochondrial fission has been considered to rescue myocardial infarction/heart failure [7]. The deletion of Mfn1/2 appears to render the heart less resistant to MPTP opening [51], whereas overexpression of Mfn1/2 prevents the opening of MPTP and reduces cell death following ischemia/reperfusion (I/R) [52, 53]. Opa1 mutation in myocardium results in mitochondrial dysfunction and increases ROS [54], while transfection of Opa1 in vivo protects mice from denervation-induced ischemic heart damage [55]. Moreover, inhibition of Drp1 significantly decreased I/R-induced mitochondrial fragmentation and cardiomyocytes apoptosis [52, 56]. Cumulative lines of evidence suggest that impaired mitochondrial dynamics are an early event in the progression of IHD that involve excessive mitochondrial fission and mitochondrial dysfunction, indicating that dynamics related proteins may be developed as new therapeutic targets against IHD.

## 4. The Translocases of Outer Membrane (Tom) Complex

The translocases of mitochondrial outer membrane (Tom) complex is a general entry gate for the importing of all mitochondrial preproteins. It comprises a central channel (formed by Tom40, Tom5, Tom6, and Tom7) and three receptors (Tom70, Tom20, and Tom22) [57]. Tom20 and Tom70 can recognize mitochondrial-targeting sequences on the precursor proteins and transfer them to Tom22 and the central channel [57, 58]. Tom20 and Tom22 mainly recognize the presequences of cleavable precursor proteins, while Tom70 typically largely recognizes the internal targeting signals of hydrophobic precursor proteins, many of which are inner membrane metabolite carriers [58]. In addition, Tom70 can also function as a docking site for cytosolic chaperones, such as Hsp70 and Hsp90, in order to receive mitochondrial proteins [59].

It has been reported that mitochondrial Tom20 was reduced by ischemia, and the maintenance of Tom20 by preconditioning confers cardioprotection via improving mitochondrial structure and function [60]. Tom20 can also restore the translocation of Cx43 to the inner mitochondrial membrane to attenuate ischemic myocardial injury [60]. Conversely, proteasome inhibitors, such as MG115, proteasome inhibitor 1, and lactacystin, lead to higher levels of Tom20 and enhance the perinuclear accumulation of mitochondria, revealing the influence of cellular redox conditions on mitochondrial import [61]. Moreover, Tom70 is obviously suppressed in hypertrophic hearts, and genetic downregulation of Tom70 worsens pathological cardiomyocyte hypertrophy. The defective mitochondrial import of Tom70-targeted Opa1 triggered intracellular oxidative stress, which led to a pathological cellular response [62]. Adenosine administration in ischemic myocardium may exhibit protection by inducing the translocation of PKC*ε* to mitochondria in a Tom70/HSP90-dependent manner [63]. However, the detailed mechanisms about Tom complex in IHD remain obscured to date, so further studies are needed.

## 5. The Other Functional Proteins in Mitochondrion

### 5.1. Mitochondrial Permeability Transition Pore (MPTP)

MPTP is a nonselective channel locating on the inner mitochondrial membrane. ROS overproduction and calcium overload can dissipate the proton electrochemical gradient and open the MPTP, leading to uncoupling of oxidative phosphorylation and further production of ROS [64, 65]. The opening of MPTP will release proapoptotic proteins to induce cell apoptosis or necrosis [13, 65]. Several studies have demonstrated that MPTP inhibition can mitigate cell loss in cardiac pathologies, including MI/R [66], heart failure [67], and diabetic cardiomyopathy [68]. Hausenloy and his colleagues revealed that inhibition of MPTP opening, during the first few minutes of reperfusion, protects myocytes from oxidative stress and finally limits infarct size [69]. However, Saotome et al. claimed that ROS-induced transient opening of MPTP protects from I/R-induced myocardium injury [70], revealing that the specific function of MPTP depends on the degree and the timing.

### 5.2. Mitochondrial Ca^2+^ Uniporter (MCU)/Mitochondrial Ca^2+^ Uptake 1 (MICU1)/MICU2

As a universal secondary messenger, Ca^2+^ plays a central role in a wide range of cellular processes, such as muscular contraction, synaptic transmission, cell migration, and cell proliferation [71]. In the heart, Ca^2+^ is central to cardiac excitation-contraction coupling and signaling networks in the regulation of pathological myocardial growth and remodeling [72]. Accumulating lines of evidence indicate that Ca^2+^ overload is linked with mitochondrial dysfunction, contractile dysfunction, and cell death [23, 73–77]. Thus, maintenance of mitochondrial Ca^2+^ homeostasis is very important for the survival of cardiomyocytes under ischemic stress [78]. MCU is an inner mitochondrial membrane channel responsible for Ca^2+^ uptake into the matrix [79]. It plays a fundamental role in the control of aerobic metabolism as well as apoptosis [80]. MCU blockade may protect the heart from hypoxia/reoxygenation injury through suppressing mitochondria-originated production of ROS [81]. Recently, MICU1 and MICU2 are identified to function as the gatekeepers of MCU by the binding of Ca^2+^ to their EF hands [82]. And MICU2's activity requires the presence of MICU1 [83]. Under basal cytosolic [Ca^2+^] condition, Ca^2+^-free MICU1 and MICU2 can inhibit MCU function; at very high cytosolic [Ca^2+^] condition, all EF hands of MICU1 and MICU2 bind Ca^2+^ to activate MCU by dissociation of MICUs from MCU complex [82]. Consistently, Mallilankaraman et al. [84] prove that MICU1 is required to preserve mitochondrial [Ca^2+^], under basal condition. In the absence of MICU1, mitochondria suffers Ca^2+^ overload, leading to excessive ROS generation. In addition, Patron et al. [85] also reveal that MICU2 largely shuts down MCU activity at low cytosolic [Ca^2+^], whereas in response to high cytosolic [Ca^2+^], MICU1 appears to stimulate Ca^2+^ uptake by MCU. Very recently, Wang et al. claim that Mfn2 overexpression reduces the expression of MICU1 and MICU2 to trigger influx of Ca^2+^ into mitochondria [86], revealing the close relationship of mitochondrial dynamics with Ca^2+^ homeostasis. However, the full functions of MICU1 and MICU2 in cardiovascular diseases remain unclear yet, emphasizing an urgent need for deep research.

### 5.3. Connexin 43 (Cx43)

Gap junction channels provide the basis for intercellular communication in cardiovascular systems, in order to keep metabolic interchange and maintain normal cardiac rhythm. In cardiomyocytes, Cx43 is the most abundant isoform. The alteration of Cx43 was observed in myocardium diseases, such as ischemia, hypertrophic cardiomyopathy, and heart failure [87]. The normal location of Cx43 at inner mitochondrial membrane is very important in the synchronization of contraction for cardiomyocytes [88, 89]. It was suggested that I/R injury is accompanied with the change of Cx43 expression [90]. Ischemic conditions can trigger Cx43 hemichannel opening, possibly mediated by the generation of ROS and nitrogen species [91]. The protection of preconditioning has been confirmed to depend on functional Cx43-formed channels [2]. Moreover, Cx43 deficiency will lead to ventricular arrhythmia which is the major cause of sudden death in heart failure. The proteasome inhibitor can be used to attenuate the degradation of Cx43 to prevent Cx43-mediated arrhythmia in heart failure [92]. Therefore, Cx43 may be developed as a potential therapeutic target for cardiovascular diseases [90].

### 5.4. Signal Transducer and Activator of Transcription 3 (STAT3)

As a transcription factor, STAT3 has been implicated to protect hearts from acute ischemic injury under stressful conditions [93], and loss of STAT3 will result in cardiomyopathy [94]. Accumulative lines of evidence also demonstrated that STAT3 serves as a protective molecule for the heart in hypertension and advanced age [95]. STAT3 is now known to be present in cardiac mitochondria and be able to regulate ROS generation and MPTP opening [96]. Wegrzyn and colleagues have proved that STAT3 deficiency can reduce the activities of complexes I and II [97]. Szczepanek et al. further revealed that mutation in the DNA binding domain of mitochondrial-targeted STAT3 can disrupt ETC activity in the heart [96]. Consistently, STAT3 transgene reduces the vulnerability of cardiac mitochondria to ischemia by restoring complex I activity and suppressing ROS generation [98]. Thus, in pathologic settings such as ischemia or early reperfusion, STAT3 may be an ideal therapeutic target to protect cardiac mitochondria.

## 6. Conclusion

**Figure 1:**
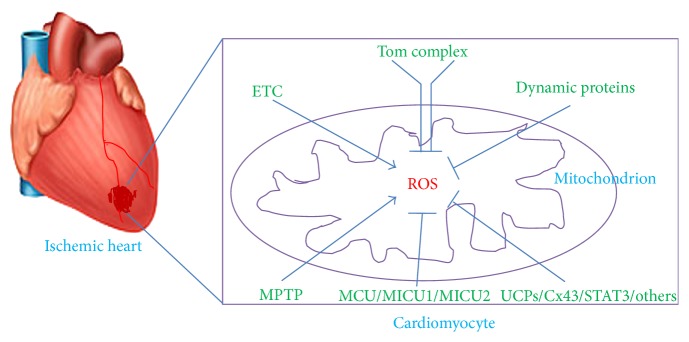
Schematic diagram indicating mitochondrial proteins' role in ROS generation in ischemic heart diseases. Under ischemic insult, the generation of ROS significantly increases in mitochondria of cardiomyocytes, which can be directly or indirectly influenced by mitochondrial functional proteins, including ETC complexes, UCPs, mitochondrial dynamic proteins, Tom complex, MPTP, MCU/MICU1/MICU2, Cx43, and STAT3. ROS: reactive oxygen species; ETC: electron transport chain; UCPs: uncoupling proteins; Tom: translocases of outer membrane; MPTP: mitochondrial permeability transition pore; MCU: mitochondrial Ca^2+^ uniporter; MICU1: mitochondrial Ca^2+^ uptake 1; MICU2: mitochondrial Ca^2+^ uptake 2; Cx43: Connexin 43; STAT3: signal transducer and activator of transcription 3.

In summary, mitochondrial functional proteins play critical roles in the production of ROS in IHD: (1) the defective ETC activity, notably of decreased activity of complex I, may form the pathological foundation for mitochondria-derived ROS overload; (2) the disruption of mitochondrial dynamics, especially depressed mitochondrial fusion, will aggravate mitochondrial ROS production; (3) Tom complex may possess important property in regulating oxidative stress, perhaps via influencing the translocation of mitochondrial proteins; (4) the other functional factors, such as MPTP, MCU/MICU1/MICU2, Cx43, and STAT3, play important roles in preserving mitochondrial integrity and function, directly or indirectly through inhibiting ROS overload (Figure 1).

In recent decades, there has been great progress in screening, identifying, and developing molecules as therapeutic targets to preserve mitochondrial integrity and prevent ROS overload. As we understand more of the distinct abnormalities occurring in the mitochondria with IHD, the goal becomes to develop new methods to mitigate the mitochondrial abnormalities. Current medicines, such beta blockers, statins, and nitrates, have improved the symptoms of IHD patients; however, these medicines' effects on mitochondrial impairment and mitochondrial functional proteins in IHD are far from clear yet. Undoubtedly, more work is needed to explore the fundamental roles of mitochondrial proteins in IHD, because they are attractive mechanistic targets for cardioprotection.
